# Additional diagnostic value of lesion visibility on diffusion-weighted imaging for differentiating BI-RADS category 4 MRI-detected breast lesions

**DOI:** 10.1007/s11604-026-01981-z

**Published:** 2026-04-10

**Authors:** Saeko Usui, Mariko Goto, Koji Sakai, Toshiyuki Okamoto, Yuka Onishi, Kei Yamada

**Affiliations:** Department of Radiology, Graduate School of Medical Science, Kyoto Prefectural University of Medicine, 465 Kajiicho, Kawaramachi Hirokoji, Kamigyoku, Kyoto 602-8566 Japan

**Keywords:** Magnetic resonance imaging, Diffusion-weighted imaging, Breast cancer, MRI-detected lesion, Dynamic contrast-enhanced MRI, Apparent diffusion coefficient

## Abstract

**Purpose:**

This study aimed to evaluate whether adding diffusion-weighted imaging (DWI) to dynamic contrast-enhanced magnetic resonance imaging (DCE-MRI) improves the differentiation between nonmalignant and malignant MRI-detected lesions.

**Materials and methods:**

A total of 36 patients with 40 Breast Imaging-Reporting and Data System (BI-RADS) category 4 MRI-detected lesions were analyzed. Two readers independently evaluated lesion visibility on two *b*-value DWIs (*b* = 800 s/mm^2^, DWI_800_ and *b* = 1500 s/mm^2^, DWI_1500_) using a five-point scale (from 0 = poor to 4 = excellent) and measured the apparent diffusion coefficient (ADC) values. DWI visibility scores and ADC values were compared between nonmalignant and malignant lesions. For the diagnostic performance analysis, lesions were classified as DWI-positive (scores 2–4, suggestive of malignancy) or DWI-negative (scores 0–1, suggestive of benignity).

**Results:**

The inter-observer agreement of the DWI visibility score was substantial for both DWI_800_ (κ = 0.923) and DWI_1500_ (κ = 0.864). The lesion visibility score was higher on DWI_1500_ than on DWI_800_. The score was significantly higher for malignant than for nonmalignant MRI-detected lesions on both DWI_800_ (reader 1: 2.74 vs. 1.57, p = 0.012; reader 2: 2.95 vs. 1.81, p = 0.020) and DWI_1500_ (reader 1: 3.11 vs 2.24, p = 0.048; reader 2: 3.00 vs. 1.95, p = 0.038). For mass lesions, both DWI_800_ and DWI_1500_ achieved 100% sensitivity and a negative predictive value for both readers, whereas all DWI-negative malignant lesions demonstrated non-mass enhancement. Overall, the mean and minimum ADC values did not differ significantly between nonmalignant and malignant lesions.

**Conclusion:**

The present findings suggest that DWI visibility provides additional value for distinguishing between nonmalignant and malignant BI-RADS category 4 MRI-detected breast lesions. Particularly for mass lesions, DWI-negative MRI-detected lesions were associated with nonmalignant pathology, indicating a potential role for DWI in reducing unnecessary biopsies.

**Supplementary Information:**

The online version contains supplementary material available at 10.1007/s11604-026-01981-z.

## Introduction

Dynamic contrast-enhanced magnetic resonance imaging (DCE-MRI) is recognized as the most sensitive imaging modality for the detection of breast cancer and is widely used in clinical practice, the diagnosis of breast lesions, and preoperative assessment of tumor extent [1]. During these examinations, additional enhancing lesions that were not previously detected on mammography or ultrasonography and are unrelated to the known index lesion may be incidentally detected; such findings are referred to as “MRI-detected lesions.” These MRI-detected lesions are reported to be identified in 13.7–41.4% of preoperative breast MRI examinations; however, because the specificity of breast MRI is relatively low, these lesions are known to have a high false-positive rate, reported to range from 44 to 64.6% [2]. Consequently, when MRI-detected lesions raise suspicion for malignancy preoperatively, additional diagnostic procedures such as biopsy are frequently required [2–5]. While essential for a definitive diagnosis, these procedures are invasive and may delay treatment of the known breast cancer. Therefore, improving the diagnostic accuracy of MRI-detected lesions using noninvasive imaging techniques could help mitigate these clinical challenges. However, such MRI-detected lesions are often small and lack distinct morphological features, making differentiation between benign and malignant lesions difficult.

Diffusion-weighted imaging (DWI) reflects tissue-specific contrast based on water diffusion, with malignant lesions typically showing more restricted diffusion and lower apparent diffusion coefficient (ADC) values as a result of higher cellularity. Regarding breast MRI, previous studies have demonstrated that malignant breast lesions show lower ADC values than benign ones, and that quantitative ADC assessment can improve differentiation between benign and malignant breast lesions, thereby reducing unnecessary biopsies when used in conjunction with DCE-MRI [6–9]. Accordingly, DWI has been proposed as a complementary sequence to improve the diagnostic accuracy of breast MRI and incorporated into routine clinical protocols in many institutions. A meta-analysis comprising 14 studies (1,140 patients and 1,276 breast lesions) found that the combined use of DCE-MRI and DWI provides superior diagnostic accuracy compared with either modality alone [8]. In addition, the Eastern Cooperative Oncology Group-American College of Radiology Imaging Network Cancer Research Group reported that applying an ADC cutoff value of 1.53 × 10^–3^ mm^2^/s reduced the number of biopsies by 20.9% [7]. However, these previous studies primarily evaluated lesions identified on conventional imaging; to our knowledge, few studies have specifically investigated MRI-detected lesions.

Given these considerations, the present study aimed to evaluate whether the addition of both qualitative DWI visibility assessment and quantitative ADC measurement to DCE-MRI improves diagnostic accuracy in distinguishing nonmalignant from malignant MRI-detected lesions, and whether this combined approach may help reduce unnecessary invasive procedures.

## Materials and methods

### Study subjects

Our institutional review board approved this retrospective study (approval No.: ERB-C-3260) and waived the requirement for written informed consent. A total of 393 consecutive patients who underwent breast MRI at 3.0 T in our institute for clinical indication from January 2020 to December 2022 were included in this study. The clinical indications for breast MRI were preoperative staging of known breast cancer (n = 184), evaluation of breast lesions detected on conventional imaging (mammography or ultrasonography) (n = 172), and assessment of response to neoadjuvant therapy (n = 37).

**Fig. 1:**
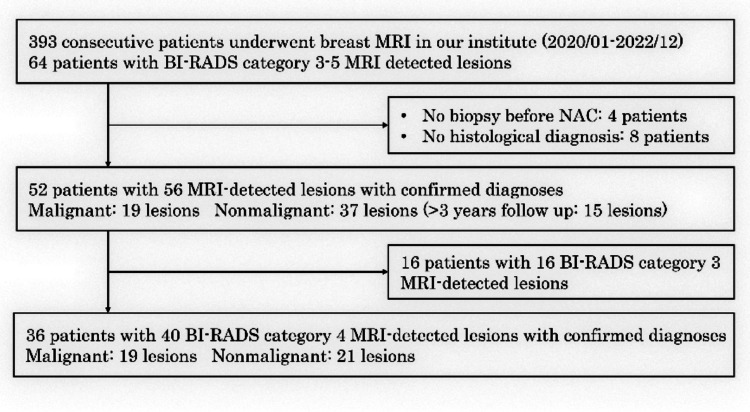
Flowchart of patient selection. No BI-RADS category 5 lesions were included in the cohort. *BI-RADS* Breast Imaging-Reporting and Data System, *NAC* neoadjuvant chemotherapy

In this study, MRI-detected lesions were defined as contrast-enhancing lesions on DCE-MRI that were documented in the breast MRI reports and distinct from known index lesions, including biopsy-proven breast cancers and lesions detected on conventional imaging. All MRI-detected lesions classified as Breast Imaging-Reporting and Data System (BI-RADS) category 3–5 were included if they were located either more than 2 cm from the index lesion within the ipsilateral breast or in the contralateral breast. For BI-RADS categorization, an experienced radiologist retrospectively reclassified lesions originally reported as BI-RADS category 3 or higher using DCE-MRI findings alone. Lesion type was determined concurrently, and lesion size was measured on the early-phase DCE-MRI slice showing the maximum lesion extent. Among 393 patients, MRI-detected lesions were found in 64 (16.3%). Among these patients, those who had received neoadjuvant chemotherapy after MRI without biopsy (n = 4), and in whom a histological diagnosis was unavailable or benignity could not be confirmed because of short-interval follow-up (n = 8) were excluded. Consequently, 52 patients with 56 MRI-detected lesions (nonmalignant: n = 37; malignant: n = 19) with confirmed diagnoses were included. Among these, no BI-RADS category 5 lesions were included, and all lesions classified as BI-RADS category 3 (n = 16) were histologically confirmed to be benign and excluded from further analysis. Ultimately, 36 patients with 40 MRI-detected lesions classified as BI-RADS category 4 (nonmalignant: n = 21; malignant: n = 19) were included in the final analysis. The patient selection process is summarized in Fig. 1.

### MRI acquisition

All patients included in the study underwent breast MRI in the prone position using a dedicated 16-channel breast coil on a 3.0-T MRI system (Magnetom Skyra; Siemens Healthcare, Erlangen, Germany). After anatomical localization, axial fat-suppressed T2-weighted images were obtained. This was followed by the acquisition of DWI in the axial plane. The parameters for the DWI were as follows: fat suppression using spectral attenuated inversion recovery; field of view at 320 mm; repetition/echo time of 10,400/61 ms; matrix size of 160 × 160 mm; slice thickness of 3 mm with 50 slices; parallel imaging factor of 2; and a total imaging duration of 2.1 min. Diffusion weighting was applied using 4 *b*-values of 0 s/mm^2^, 200 s/mm^2^, 800 s/mm^2^, and 1500 s/mm^2^. Each acquisition was performed three times, and the resulting signals were averaged to improve the signal-to-noise ratio (SNR).

Subsequently, DCE-MRI was performed using a three-dimensional fat-suppressed volume-interpolated breath-hold sequence. A gadolinium-based contrast agent (Dotarem; Guerbet, Villepinte, France) was intravenously administered at a dose of 0.1 mmol/kg body weight at a rate of 2 mL/s, followed by a 20 mL saline flush using an automatic injector. The parameters for the DCE-MRI were as follows: field of view of 320 mm; repetition/echo times of 3.3/1.4 ms; flip angle of 15°; matrix size of 352 × 352 mm with 144 slices; parallel imaging factor of 3; three contrast-enhanced acquisitions centered at 0, 90, and 300 s; and a total imaging duration of 60 s.

### DWI evaluation/visibility scoring

The visual assessment of MRI-detected lesions of two *b*-value DWIs (*b* = 800 s/mm^2^, DWI_800_ and *b* = 1500 s/mm^2^, DWI_1500_) was independently performed by two radiologists: reader 1, a board-certified radiologist with 17 years of experience in breast MRI, and reader 2, a radiology resident with 4 years of experience. The readers were informed only of the location of MRI-detected lesions and blinded to the pathological results, but were allowed to refer to the DCE-MRI during evaluation. Lesion visibility on DWI was qualitatively assessed using a five-point scale, which was pre-standardized by both readers. The scale was defined as follows: a score of 0 indicated the lesion was isointense to the background fibroglandular tissue (FGT) and not distinguishable; 1, faintly visible only with reference to DCE-MRI; 2, visible on DWI alone but with poor contrast against FGT; 3, clearly visible with moderate contrast against FGT; and 4, distinctly visible with excellent contrast against FGT. Representative images for all DWI visibility scores are provided in Supplementary Figure S1 (Online Resource 1). Each reader was allowed to independently adjust the window width and window level when assigning the scores.

### DWI evaluation/ADC measurement

ADC measurements were independently performed by two readers concurrently with the DWI visibility assessment. Two ADC maps were generated from *b*-values of 0–800 and 0–1500 s/mm^2^, hereafter referred to as ADC_0–800_ and ADC_0–1500_, respectively. For each reader, the slice showing the highest signal intensity of the lesion was selected on each DWI, specifically on DWI_800_ and DWI_1500_, and a region of interest (ROI) was manually placed to cover the lesion on an interactive workstation (Aquarius iNtuition; TeraRecon, Inc., Foster City, CA, USA), without extending beyond its margins, with reference to DCE-MRI. This ROI was then copied onto the corresponding ADC maps to measure mean and minimum ADC_0–800_ and ADC_0–1500_ values, respectively. Lesions that were not visible on DWI (DWI visibility score 0) were excluded from the ADC measurements.

### Statistical analysis

All statistical analyses were performed using R software 4.2.1 (R Foundation for Statistical Computing, Vienna, Austria) and JMP Pro version 14.0.0 (SAS Institute, Inc., Cary, NC, USA). The characteristics of both the patients and lesions were compared in each group using the chi-square test if the data were qualitative, and by the Wilcoxon rank-sum test if the characteristics were quantitative. To compare DWI visibility scores between the two *b*-values (DWI_800_ vs. DWI_1500_), the Wilcoxon signed-rank test was used. Additionally, comparisons of DWI visibility scores and ADC values between nonmalignant and malignant lesions were performed using the Wilcoxon rank-sum test.

Inter-reader agreement for DWI visibility scores was assessed using weighted kappa statistics interpreted as follows: 0–0.2, slight; 0.21–0.4, fair; 0.41–0.6, moderate; 0.61–0.8, substantial; and 0.81–1.0, almost perfect. Agreement for ADC measurements was evaluated using the intraclass correlation coefficient (ICC), with values < 0.5 considered poor, 0.5–0.75 moderate, 0.75–0.9 good, and > 0.9 excellent.

To evaluate the diagnostic performance of DWI visibility, lesions were classified as DWI-positive (score 2–4, suggestive of malignancy) or DWI-negative (score 0–1, suggestive of benignity). Based on this classification, sensitivity, specificity, the positive predictive value (PPV), and negative predictive value (NPV) were calculated for all lesions, mass lesions, and non-mass enhancement (NME) lesions.

## Results

### Patient and lesion characteristics

Table 1 summarizes the characteristics of the patients and MRI-detected lesions. Among the included cases, 18 lesions were benign and 19 were malignant. Histological diagnoses were obtained from either biopsy or surgical specimens of known breast cancer. Of the 18 benign lesions, five were not histologically confirmed but remained stable during imaging follow-up exceeding 3 years, including mammography and ultrasonography. In addition, three lesions were classified as atypical on biopsy; however, none demonstrated progression to malignancy over the same follow-up period. Accordingly, 21 lesions were categorized as nonmalignant and 19 as malignant, thereby defining two groups. The benign or malignant status of the index lesion differed significantly between the MRI-detected nonmalignant and malignant groups (*p* = 0.021), and no additional malignant lesion was detected on MRI when the index lesion was benign. Menopausal status also differed significantly between these groups, with a higher proportion of postmenopausal patients in the malignant group (*p* = 0.043). Among the premenopausal patients (n = 13), MRI was performed during the follicular phase (days 7–14) in 3 patients, during other phases in 7 patients, and menstrual cycle information was unavailable in 2 patients. The size of the MRI-detected lesions was generally small in both the nonmalignant (8.0 ± 2.4 mm) and malignant groups (7.8 ± 3.0 mm), with 83% (n = 33/40) of all lesions measuring < 1 cm. No significant differences were observed between nonmalignant and malignant MRI-detected lesions with respect to lesion location (ipsilateral vs. contralateral), size, or type (mass vs. NME) as assessed on DCE-MRI.

**Table 1:** Patient demographics of 36 women and pathologic findings on 40 MRI-Detected lesions

Characteristics	MRI-detected lesions			
	All (n = 40)	Non-malignant (n = 21)	Malignant (n = 19)	P-value
Age (years)^†^	56.6 ± 12.6	52.9 ± 13.4	61.3 ± 10.1	0.069^b^
Menopausal status				
Premenopausal	14 (35)	11 (52)	3 (16)	0.043^a*^
Postmenopausal	25 (63)	10 (48)	15 (79)	
Unknown	1 (3)	0	1 (5)	
MRI indication				
Preoperative staging	19 (48)	10 (48)	9 (47)	1.000^a^
Evaluation of breast lesion detected by conventional imaging	21 (53)	11 (52)	10 (53)	
Background parenchymal enhancement				
minimal/mild	33 (83)	16 (76)	17 (89)	0.412^a^
moderate/marked	7 (18)	5 (24)	2 (11)	
Final diagnoses of index lesions				
Malignant	34 (85)	15 (71)	19 (100)	0.021^a^*
Benign	6 (15)	6 (29)	0 (0)	
Location of MRI-detected lesions				
Ipsilateral of index lesion	16 (40)	7 (33)	9 (47)	0.520^a^
Contralateral of index lesion	24 (60)	14 (67)	10 (53)	
Lesion size on DCE-MRI (mm)^†^	7.9 ± 2.7	8.0 ± 2.4	7.8 ± 3.0	0.464^b^
> 1 cm	7 (18)	4 (19)	3 (16)	1.000^a^
≤ 1 cm	33 (83)	17 (81)	16 (84)	
Lesion type on DCE-MRI				
Mass	21 (53)	10 (48)	11 (58)	0.545^a^
Non-mass enhancement	19 (48)	11 (52)	8 (42)	
Histological diagnosis				
Invasive ductal carcinoma	–	–	9 (47)	–
Ductal carcinoma in situ	–	–	6 (32)	–
Invasive lobular carcinoma	–	–	3 (15)	–
Tubular carcinoma	–	–	1 (5)	–
Fibrocystic change	–	4 (19)	–	–
Fibrosis	–	2 (10)	–	–
Radial scar	–	2 (10)	–	–
Mastitis	–	1 (5)	–	–
Ductal adenoma	–	1 (5)	–	–
Usual ductal hyperplasia	–	1 (5)	–	–
Intraductal papilloma	–	2 (10)	–	–
Atypical lesion	–	3 (14)	–	–
No histological diagnosis^‡^	–	5 (24)	–	–

− Unless otherwise indicated, data are numbers of lesions and data in parentheses are percent

^†^ Data are mean ± standard deviation

^‡^ Observed for more than 3 years with no clinical change

^a^ P*-*values for differences were calculated using the chi-square test

^b^ P-values for differences were calculated using the Wilcoxon rank-sum test

*Denotes statistical significance (p < 0.05)

DCE, dynamic contrast-enhanced

### DWI visibility

Table 2 presents a comparison of the mean and standard deviation visibility scores between the nonmalignant and malignant groups for each DWI. Regarding lesion diagnosis, both readers assigned significantly higher scores to malignant than to nonmalignant lesions at both DWI_800_ and DWI_1500_.

**Table 2:** Visibility score by lesion diagnosis for two b-values of DWI

	Reader 1			Reader 2		
	Lesion diagnosis		^a^P-value	Lesion diagnosis		^a^P-value
	Nonmalignant (n = 21)	Malignant (n = 19)		Nonmalignant (n = 21)	Malignant (n = 19)	
DWI_800_	1.57 ± 1.33	2.74 ± 1.37	0.012*	1.81 ± 1.54	2.95 ± 1.18	0.020*
DWI_1500_	2.24 ± 1.51	3.11 ± 1.41	0.048*	1.95 ± 1.72	3.00 ± 1.29	0.038*
^b^P-value	< 0.001*	0.109		0.362	0.875	

—Data are expressed as the mean ± standard deviation

DWI, diffusion-weighted imaging; DWI_800_, *b*-value of 800 s/mm^2^ DWI; DWI_1500_, *b*-value of 1500 s/mm^2^ DWI

^a^ P-values between the nonmalignant and malignant groups were calculated using the Wilcoxon rank-sum test

^b^ P-values between DWI_800_ and DWI_1500_ were calculated the Wilcoxon signed-rank test

*Denotes statistical significance (p < 0.05)

Between the two DWIs, both readers assigned higher mean visibility scores to lesions on DWI_1500_ than on DWI_800_ in both groups, with the difference reaching statistical significance for nonmalignant lesions evaluated by reader 1 (Table 2, *p* < 0.001). The number of lesions for each DWI visibility score is provided in Supplementary Table S1 (Online Resource 1). Score increases from DWI_800_ to DWI_1500_ were observed in 12 nonmalignant and 6 malignant lesions for Reader 1, and in 6 nonmalignant and 5 malignant lesions for Reader 2.

The inter-reader agreement for the visibility score was almost perfect for both DWI_800_ (κ = 0.923) and DWI_1500_ (κ = 0.864).

### Diagnostic performance of DWI visibility

The diagnostic performance of each DWI for each reader, presented for all lesions and according to DCE-MRI lesion type, is summarized in Table 3.

**Table 3:** Diagnostic performance of DWI visibility scores for MRI-detected lesions on DWI_800_ and DWI_1500_

	Reader 1			Reader 2		
	All lesions (n = 40)	Mass (n = 21)	NME (n = 19)	All lesions (n = 40)	Mass (n = 21)	NME (n = 19)
*DWI*_*800*_						
Sensitivity (%)	73.7 (14/19)	100.0 (11/11)	37.5 (3/8)	89.5 (17/19)	100.0 (11/11)	75.0 (6/8)
Specificity (%)	47.6 (10/21)	40.0 (4/10)	54.5 (6/11)	47.6 (10/21)	40.0 (4/10)	54.5 (6/11)
NPV (%)	66.7 (10/15)	100.0 (4/4)	54.5 (6/11)	83.3 (10/12)	100.0 (4/4)	75.0 (6/8)
PPV (%)	56.0 (14/25)	64.7 (11/17)	37.5 (3/8)	60.7 (17/28)	64.7 (11/17)	54.5 (6/11)
*DWI*_*1500*_						
Sensitivity (%)	84.2 (16/19)	100.0 (11/11)	62.5 (5/8)	89.5 (17/19)	100.0 (11/11)	75.0 (6/8)
Specificity (%)	42.9 (9/21)	40.0 (4/10)	45.5 (5/11)	47.6 (10/21)	50.0 (5/10)	45.5 (5/11)
NPV (%)	75.0 (9/12)	100.0 (4/4)	62.5 (5/8)	83.3 (10/12)	100.0 (5/5)	72.4 (5/7)
PPV (%)	57.1 (16/28)	64.7 (11/17)	45.5 (5/11)	60.7 (17/28)	68.9 (11/16)	50.0 (6/12)

— Data are expressed as percentages. Data in parentheses are the number of lesions. Sensitivity, specificity, NPV, and PPV were calculated by classifying lesions with DWI scores of 0–1 as DWI-negative (suggestive of benignity) and those with scores of 2–4 as DWI-positive (suggestive of malignancy)

NPV, negative predictive value; PPV, positive predictive value; NME, non-mass enhancement; DWI, diffusion-weighted imaging; DWI_800_, *b*-value of 800 s/mm^2^ DWI; DWI_1500_, *b*-value of 1500 s/mm^2^ DWI

For all lesions, reader 2 demonstrated comparable sensitivity and NPV between DWI_800_ and DWI_1500_ (sensitivity 89.5% and NPV 83.3% for both), whereas reader 1 exhibited higher sensitivity and NPV on DWI_1500_ (sensitivity 84.2% and NPV 75.0%) compared with DWI_800_ (sensitivity 73.7% and NPV 66.7%). In the analysis according to lesion type, the difference was mainly due to NME lesions; for NME lesions, reader 2 showed similar sensitivity and NPV between DWI_800_ and DWI_1500_, whereas reader 1 exhibited lower sensitivity and NPV on DWI_800_ (sensitivity 37.5% and NPV 54.4%) compared with DWI_1500_ (sensitivity 62.5% and NPV 62.5%).

**Fig. 2:**
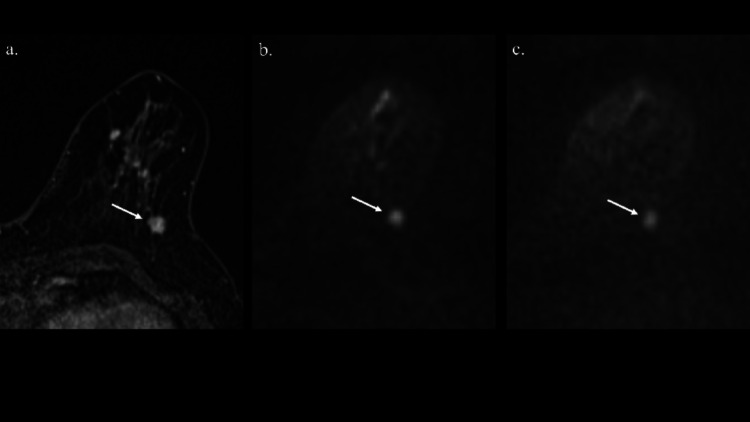
A 70-year-old woman underwent preoperative MRI for invasive ductal carcinoma in the right breast. On early-phase DCE-MRI, an 8.1-mm MRI-detected mass lesion was observed in the left breast (contralateral to the known breast cancer) (a, arrow) and assessed as BI-RADS category 4. On both DWI (b, DWI_800_ and c, DWI_1500_), the lesion was clearly visualized with high contrast against the fibroglandular tissue (b, c, arrow). Both readers assigned a DWI visibility score of 4 on both DWI. On MRI-targeted ultrasonography, the lesion was depicted as a 6.6-mm mass and was diagnosed as invasive ductal carcinoma by ultrasound-guided core needle biopsy (DWI true-positive)

**Fig. 3:**
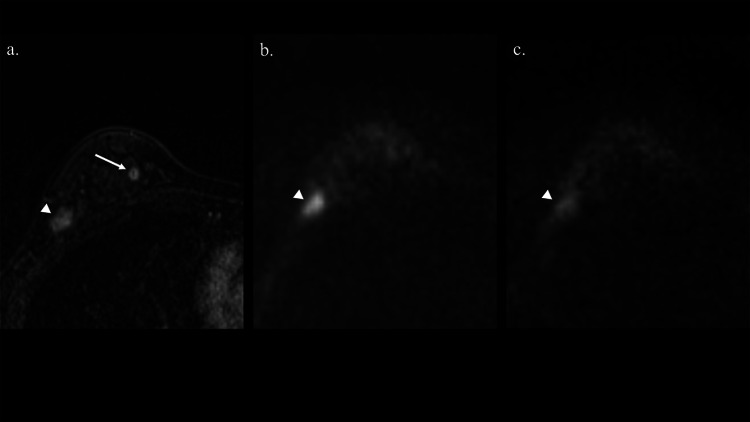
A 53-year-old woman underwent breast MRI for evaluation of a lesion detected on mammography and ultrasonography in the right breast. The index lesion (a–c; arrowheads) was pathologically diagnosed as fibroadenoma. On early-phase DCE-MRI, a 5.2-mm MRI-detected mass was identified in the ipsilateral breast (a, arrow) and assessed as BI-RADS category 4. On both DWI (b, DWI_800_ and c, DWI_1500_), the lesion was isointense to the surrounding fibroglandular tissue and not visually distinguishable (b, c, arrow). Both readers assigned a DWI visibility score of 0 on both DWI. On MRI-targeted ultrasonography, the lesion was depicted as a 6-mm mass and was diagnosed as fibrosis by ultrasound-guided core needle biopsy (DWI true-negative)

**Fig. 4:**
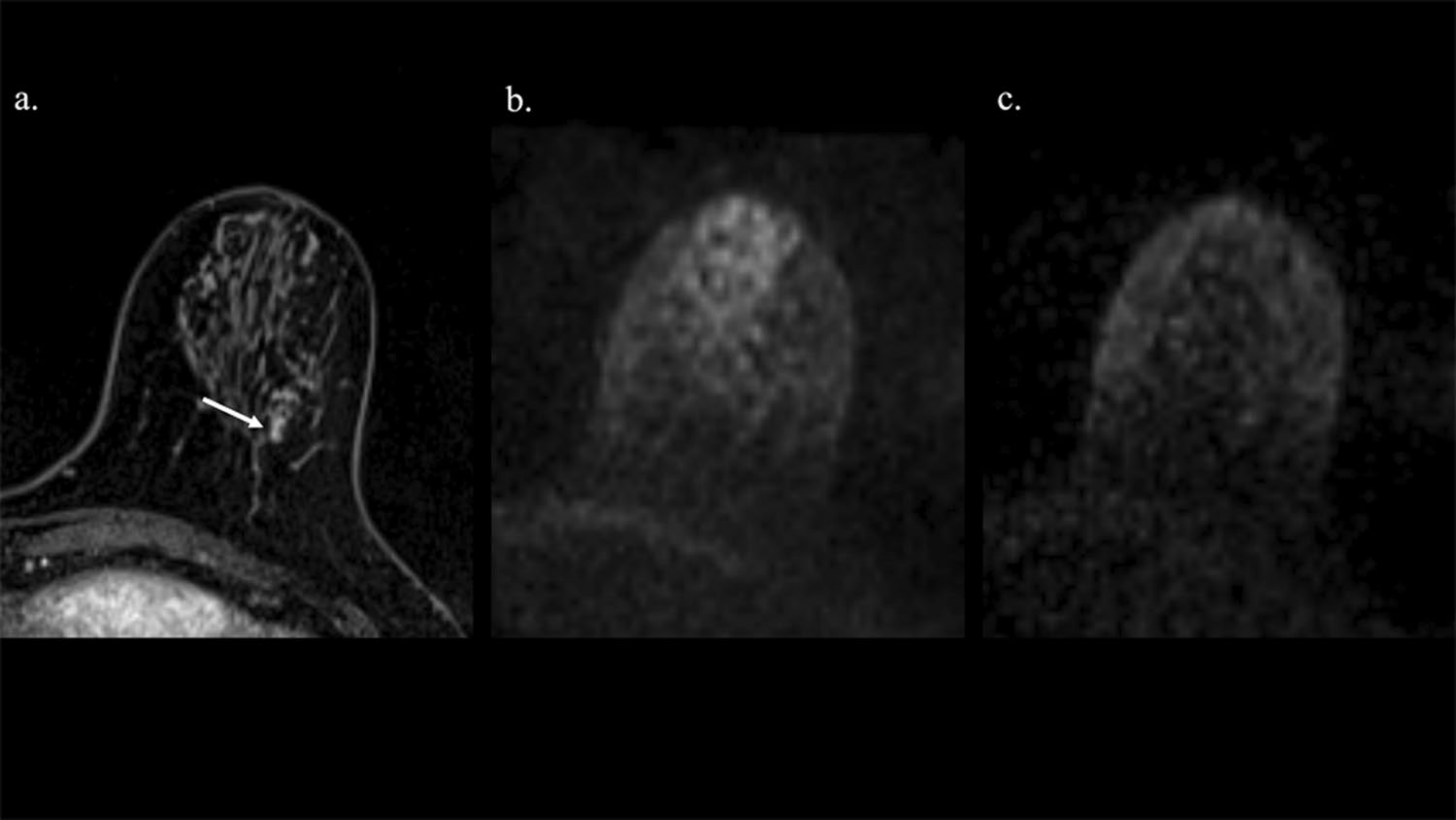
A 65-year-old woman underwent preoperative MRI for evaluation of ductal carcinoma in situ in the right breast. On early-phase DCE-MRI, an 8.2-mm non-mass enhancement (NME) was detected in the left breast (contralateral to the known breast cancer) (a, arrow) and assessed as BI-RADS category 4. On both DWI (b, DWI_800_ and c, DWI_1500_), the lesion was isointense to the surrounding fibroglandular tissue and not visually distinguishable. Both readers assigned a DWI visibility score of 0 on both DWI. On MRI-targeted ultrasonography, the lesion was depicted as an approximately 5-mm hypoechoic area and was diagnosed as invasive lobular carcinoma by ultrasound-guided vacuum-assisted biopsy (DWI false-negative)

For mass lesions, both DWI_800_ and DWI_1500_ demonstrated 100% sensitivity and NPV for both readers, and all malignant lesions that were DWI-negative for both readers were NME lesions. Representative cases of DWI true-positive and true-negative masses and of DWI false-negative NME are presented in Figs. 2, 3 and 4, respectively. On DWI_800_, the malignant NME lesions evaluated as DWI-negative included ductal carcinoma in situ (DCIS) (n = 3), invasive ductal carcinoma (IDC) (n = 1), and invasive lobular carcinoma (ILC) (n = 1) by reader 1, and DCIS (n = 1) and ILC (n = 1) by reader 2. On DWI_1500_, DWI-negative NME lesions included DCIS (n = 2) and ILC (n = 1) by reader 1, and DCIS (n = 1) and ILC (n = 1) by reader 2.

### ADC measurement

Table 4 summarizes the mean and minimum ADC for each lesion type on DCE-MRI, presented separately for each reader. The numbers of lesions excluded from ADC measurement (lesions with a DWI visibility score of 0) were as follows. For Reader 1, 6 nonmalignant and 1 malignant lesions were excluded on DWI_800_, and 3 nonmalignant and 2 malignant lesions on DWI_1500_. For Reader 2, 6 nonmalignant and 1 malignant lesions were excluded on DWI_800_, and 7 nonmalignant and 1 malignant lesions on DWI_1500_.

**Table 4:** ADC Values in MRI-detected Non-malignant and Malignant Lesions

Reader	ADC_0-800_ (× 10^–3^ mm^2^/s)							ADC_0-1500_ (× 10^–3^ mm^2^/s)						
	Lesion type	ADC parameter	Non malignant	(n)	Malignant	(n)	P-value	Lesion type	ADC parameter	Non malignant	(n)	Malignant	(n)	P-value
Reader 1	All (n = 33)	ADC_mean_	1.14 ± 0.30	(15)	0.94 ± 0.23	(18)	0.065	All (n = 35)	ADC_mean_	0.98 ± 0.24	(18)	0.82 ± 0.25	(17)	0.074
		ADC_min_	0.89 ± 0.25		0.72 ± 0.27		0.070		ADC_min_	0.71 ± 0.29		0.58 ± 0.29		0.114
	Mass (n = 20)	ADC_mean_	1.17 ± 0.36	(9)	0.90 ± 0.21	(11)	0.040*	Mass (n = 20)	ADC_mean_	1.00 ± 0.29	(9)	0.79 ± 0.23	(11)	0.111
		ADC_min_	0.95 ± 0.27		0.66 ± 0.23		0.023*		ADC_min_	0.69 ± 0.37		0.58 ± 0.27		0.323
	NME (n = 13)	ADC_mean_	1.10 ± 0.23	(6)	1.00 ± 0.27	(7)	0.402	NME (n = 15)	ADC_mean_	0.96 ± 0.20	(9)	0.87 ± 0.29	(6)	0.397
		ADC_min_	0.83 ± 0.23		0.82 ± 0.32		1.000		ADC_min_	0.73 ± 0.20		0.58 ± 0.35		0.397
Reader 2	All (n = 33)	ADC_mean_	1.06 ± 0.23	(15)	0.95 ± 0.22	(18)	0.164	All (n = 32)	ADC_mean_	0.93 ± 0.21	(14)	0.78 ± 0.24	(18)	0.065
		ADC_min_	0.82 ± 0.28		0.67 ± 0.28		0.143		ADC_min_	0.53 ± 0.29		0.57 ± 0.30		0.624
	Mass (n = 19)	ADC_mean_	1.07 ± 0.25	(8)	0.90 ± 0.22	(11)	0.076	Mass (n = 19)	ADC_mean_	0.92 ± 0.26	(8)	0.76 ± 0.23	(11)	0.127
		ADC_min_	0.84 ± 0.31		0.62 ± 0.27		0.127		ADC_min_	0.52 ± 0.33		0.50 ± 0.28		0.962
	NME (n = 14)	ADC_mean_	1.04 ± 0.22	(7)	1.04 ± 0.21	(7)	0.898	NME (n = 13)	ADC_mean_	0.93 ± 0.15	(6)	0.78 ± 0.27	(7)	0.284
		ADC_min_	0.80 ± 0.28		0.75 ± 0.29		1.000		ADC_min_	0.54 ± 0.26		0.67 ± 0.31		0.289

—Data are mean ± standard deviation. Numbers in parentheses indicate the number of lesions

ADC, apparent diffusion coefficient; NME, non-mass enhancement

P-values for differentiation were calculated using the Wilcoxon rank-sum test

*Denotes statistical significance (p < 0.05)

No significant differences were observed between nonmalignant and malignant lesions for any ADC values for all lesions. In the analysis of only mass lesions, reader 1 demonstrated that the malignant group had a significantly lower mean ADC_0–800_ value of 0.90 ± 0.21 mm^2^/s and a minimum ADC_0–800_ value of 0.66 ± 0.23 mm^2^/s compared with the nonmalignant group, which had mean and minimum values of 1.17 ± 0.36 mm^2^/s and 0.95 ± 0.27 mm^2^/s, respectively. By contrast, reader 2 did not show significant differences between the two groups. No significant differences were found for ADC_0–1500_ in the mass lesion group assessed by reader 2, or in the analysis including NME lesions.

The ICC for the ADC measurement was good for ADC_0–800_ (mean, 0.867 and minimum, 0.847). The ICC indicated good agreement for mean ADC_0–1500_ (0.774), while the minimum ADC_0–1500_ indicated moderate agreement (0.666).

## Discussion

To evaluate the diagnostic utility of DWI in MRI-detected lesions, we assessed both lesion visibility on DWI as a qualitative parameter and the ADC value as a quantitative parameter. Our results demonstrated that DWI_1500_ provided superior visibility for MRI-detected lesions compared with DWI_800_, providing stable diagnostic performance independent of the readers. Furthermore, the diagnostic performance based on lesion visibility was highly effective for distinguishing nonmalignant from malignant MRI-detected mass lesions; however, for NME lesions, malignant lesions could not be reliably excluded even when DWI was negative. In addition, the diagnostic utility of ADC measurements appears to be limited.

In this study, MRI-detected lesions were identified in 16.3% of cases, consistent with prior reports (13.7%–41.4% of preoperative breast MRI) [2]. All BI-RADS 3 lesions in our cohort were benign, and because no category 5 lesions were included, the final cohort comprised only BI-RADS 4 lesions, with a malignancy rate of 47.5%. Although Lee et al. [3] reported a lower malignancy rate of 17.8% for MRI-detected BI-RADS 4 lesions in a preoperative breast MRI cohort, this difference may reflect variations in the composition of BI-RADS 4 lesions between study populations. The probability of malignancy for BI-RADS 4 lesions ranges widely, from 2 to 95% [10]; previous studies likely included a higher proportion of lesions with a lower likelihood of malignancy, whereas our cohort may have contained a larger proportion of lesions with a higher likelihood of malignancy. Nevertheless, the malignancy rate observed in this study falls within the malignancy probability range defined for BI-RADS category 4.

Improving the diagnostic accuracy of BI-RADS category 4 MRI-detected lesions in clinical breast MRI is important to avoid unnecessary biopsies and prevent delays in the treatment of known breast cancers. DWI/ADC is often incorporated into routine breast MRI; although previous studies have investigated its supplemental diagnostic utility for BI-RADS 4 lesions, there has been little evaluation specifically for MRI-detected BI-RADS 4 lesions [6]. In this study, the visibility of MRI-detected BI-RADS 4 lesions was consistently higher on DWI_1500_ than on DWI_800_, independent of the reader. This finding is consistent with previous reports. For example, Bickel et al. [11] reported that DWI using *b*-values between 1200 and 1800s/mm^2^ provides superior image quality and lesion visibility for breast MRI, and the Diffusion-Weighted Magnetic Resonance Imaging Screening Trial (DWIST) study used a *b*-value of 1200 s/mm^2^ DWI for lesion visualization [12]. Although higher *b*-values reduce the SNR, lesion visibility improves because stronger suppression of FGT signals increases the lesion-to background contrast [11, 13, 14]. In line with these findings, our results suggest that high *b*-value DWI may similarly improve the detection of MRI-detected lesions.

In this study, we found that malignant MRI-detected lesions exhibited higher DWI visibility scores than nonmalignant lesions, and that mass lesions demonstrated perfect sensitivity and NPV irrespective of the reader or DWI *b*-value. These results support previous findings by Baltzer et al. [15], who reported that non-contrast breast MRI for breast lesions may offer lower visibility compared with contrast-enhanced MRI, but lesions that were not visible on DWI tended to be benign. Accordingly, our results suggest that MRI-detected BI-RADS 4 mass lesions that are DWI-negative may reasonably be considered nonmalignant without missing any malignancies, regardless of the reader’s level of experience. Based on this approach, four of 10 (40%) nonmalignant mass lesions in our cohort could have avoided unnecessary biopsy.

By contrast, the diagnostic performance for MRI-detected NME lesions was lower than that for mass lesions across all *b*-values and readers. Previous studies have also demonstrated the lower diagnostic accuracy of DWI for NME lesions than for mass lesions, with one report indicating that 31% of NME lesions were not visible on DWI [16–18]; the present findings are consistent with these previous observations. Particularly, the inter-reader discrepancy in sensitivity for NME was more pronounced at DWI_800_ than at DWI_1500_, indicating the limited reliability of the assessment. In this study, lesion conspicuity on DWI was evaluated based on signal intensity relative to FGT; NME lesions often show low inherent signal conspicuity relative to FGT, particularly on DWI_800_, where the suppression of background signals is weaker than on DWI_1500_. Because DWI assessment was performed with reference to the corresponding findings on DCE-MRI, reflecting routine clinical interpretation, the evaluation of these low-contrast lesions may have been influenced more by the DCE-MRI findings, which may have contributed to inter-reader differences in interpretation. By contrast, DWI_1500_ improved lesion conspicuity, potentially reducing inter-reader variability and providing a more stable basis for evaluating NME lesions.

When examining malignant lesions that were interpreted as DWI-negative by each reader on both DWI, all of these lesions were NME. Further analysis of the histopathology of these malignant NME lesions revealed that those remaining invisible even on DWI_1500_ were DCIS and ILC. By contrast, all IDC presenting as NME were visible by both readers on DWI_1500_. It is generally recognized that the visibility of DCIS on DWI is lower than that of invasive carcinomas, reflecting its lower cellular density and intraductal growth pattern [11, 17, 18]. Similarly, ILC has been reported to show lower visibility on DWI compared with other types of invasive carcinoma [19]. Taken together, these findings suggest that while DWI_1500_ may allow more reliable detection of IDC manifesting as NME regardless of the reader, malignant lesions could not be reliably excluded even when DWI_1500_ was negative. The presence of inherently low-visibility lesions such as DCIS and ILC within the NME category likely limits the diagnostic performance of DWI for BI-RADS 4 NME lesions.

In the evaluation of breast lesions, ADC values have been extensively investigated as potential biomarkers for lesion characterization, and the European Society of Breast Imaging (EUSOBI) recommends the use of mean ADC measurements [20]. Previous studies have reported that minimum ADC values may reflect the most aggressive component of a tumor and can be useful in differentiating between benign and malignant lesions [21–23]. In the present study, significant differences in both mean and minimum ADC values between nonmalignant and malignant lesions were observed only for mass lesions evaluated by reader 1 using ADC_0–800_. No such differences were found with ADC_0–1500_, in the mass lesion group assessed by reader 2 or in the analyses including all lesions and NME lesions. Furthermore, in this study, ADC_0–1500_ demonstrated lower ICCs than ADC_0–800_, indicating limited inter-reader reproducibility at higher *b*-values.

Although high *b*-value DWI has been reported to enhance the visibility of breast cancer, both EUSOBI guidelines [20] and the DWIST study [12] recommend using a *b* = 800 s/mm^2^ as the standard for ADC measurement, balancing specificity with an acceptable SNR. The present findings are consistent with these recommendations; however, even at *b* = 800 s/mm^2^, stable ADC measurements may not be achievable for MRI-detected lesions, suggesting limited practical utility in such cases. Previous studies have also reported the limited reliability of ADC measurements in small and NME lesions, with contributing factors likely including the infiltrative growth pattern of NME lesions, inadvertent inclusion of normal breast tissue in large ROIs, and partial volume effects [7, 16–18, 24–28]. Wan et al. [29] demonstrated that the sensitivity and specificity of ADC measurements decrease in lesions < 1 cm, which supports our findings, as 83% of the lesions in our cohort were < 1 cm in size. These technical challenges may also explain the inter-reader differences observed in ADC_0-800_ in the present study, as difficulty in ADC measurements for small and NME lesions likely resulted in variability in ROI placement between readers. Consistent with these observations, the present study suggests that although high *b*-value DWI improves lesion visibility, its application for reliable ADC measurement remains limited for small or NME lesions.

In this study, we demonstrated that adding an assessment of lesion visibility on DWI to DCE-MRI for BI-RADS category 4 MRI-detected mass lesions contributed to the differentiation between nonmalignant and malignant lesions and may help reduce the number of unnecessary biopsies. BI-RADS category 4 encompasses lesions with a wide range of malignancy risk, and the BI-RADS v2025 Manual incorporates subcategory classification of category 4 lesions for MRI assessment, enabling refined risk stratification [30]. However, these subcategories lack objective classification criteria. Several previous studies have investigated BI-RADS category 4 subcategorization using scoring systems based on DCE-MRI alone or in combination with T2-weighted imaging and/or DWI/ADC [31–35]. Our findings may provide fundamental insights into the role of DWI in developing more objective approaches to BI-RADS category 4 subcategorization. Further studies with larger cohorts are warranted.

This study has several limitations. First, it was conducted at a single institution with a relatively small sample size, which may limit the generalizability of the findings. 

In particular, for ADC measurements, lesions with poor visibility on DWI were excluded from the analysis, resulting in a smaller sample size, which may have introduced a selection bias, as lesions with low DWI visibility tended to be nonmalignant in this cohort and were therefore underrepresented in the ADC analysis. In addition, the limited and differing number of lesions available for ADC measurement between readers may also have influenced the results. Future studies with a larger cohort are warranted to validate these findings further. Second, all MRI examinations were performed for specific clinical indications, including the evaluation of lesions detected by conventional imaging and preoperative staging. This may have introduced selection bias into the cohort. Third, although MRI-detected lesions were re-evaluated by an experienced radiologist using DCE-MRI findings alone, the characteristics of the index lesion may have influenced the reassessment of BI-RADS categories for MRI-detected lesions. Fourth, although the contrast on DWI can be affected by factors such as breast tissue volume and background T2 signals, these variables were not assessed in the present study, which may have influenced the lesion visibility and ADC measurements.

In conclusion, qualitative assessment of DWI visibility is useful for distinguishing malignant from nonmalignant MRI-detected BI-RADS 4 lesions. For mass lesions, DWI-negative findings showed 100% sensitivity and NPV, suggesting that such lesions may be considered nonmalignant, potentially enabling the avoidance of unnecessary biopsies. Although DWI_1500_ improved lesion visibility and reduced inter-reader variability—particularly for NME—DWI-negative findings in NME lesions cannot reliably exclude malignancy. In addition, ADC measurements, especially for small or NME lesions, showed limited reproducibility. These results support the value of DWI as a complementary tool in preoperative breast MRI for the evaluation of BI-RADS category 4 MRI-detected lesions, and highlight the need for careful assessment of NME lesions and further investigations in larger cohorts.

## Supplementary Information

Below is the link to the electronic supplementary material.

Supplementary Material 1.
