# Supplementary material for: Additional diagnostic value of lesion visibility on diffusion-weighted imaging for differentiating BI-RADS category 4 MRI-detected breast lesions
Source: Jpn J Radiol. 2026 Apr 10;44(8):1382–93. doi: 10.1007/s11604-026-01981-z (PMC13400719; doi:10.1007/s11604-026-01981-z)
Supplement: Supplementary file 1 — Supplementary Material 1. [file 11604_2026_1981_MOESM1_ESM.pdf]

Additional Diagnostic Value of Lesion Visibility on Diffusion-Weighted Imaging for Differentiating BI-RADS Category 4 MRI-Detected Breast Lesions.
Japanese Journal of Radiology
S Usui, M Goto, et al. Corresponding author: M Goto (gomari@koto.kpu-m.ac.jp).
Department of Radiology, Kyoto Prefectural University of Medicine

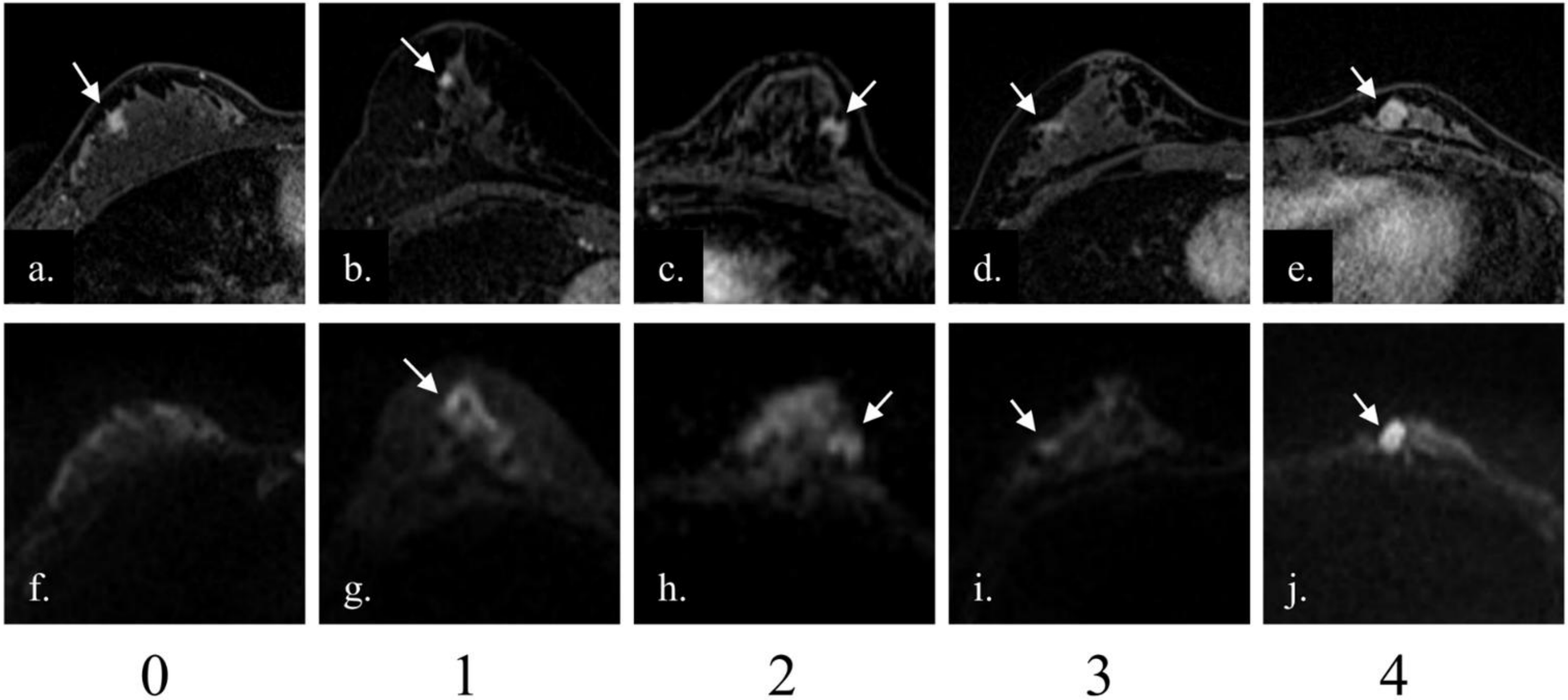

Supplementary Figure S1

Representative cases illustrating DWI visibility scores. Each MRI-detected lesion identified on early-phase DCE-MRI (a–e, arrows) and the corresponding DWI_{800} images (f–j) are arranged from left to right according to DWI visibility scores, ranging from 0 to 4.

Supplementary Table S1: Number of MRI-detected Lesions by DWI Visibility Score

DWI Visibility Score	Reader 1				Reader 2			
	DWI_{800}		DWI_{1500}		DWI_{800}		DWI_{1500}	
	Nonmalignant (n=21)	Malignant (n=19)	Nonmalignant (n=21)	Malignant (n=19)	Nonmalignant (n=21)	Malignant (n=19)	Nonmalignant (n=21)	Malignant (n=19)
0	6	1	3	2	6	1	7	1
1	4	4	6	1	4	1	3	1
2	6	2	1	2	3	4	1	6
3	3	4	5	2	4	5	4	0
4	2	8	6	12	4	8	6	11

Note.—Numbers indicate the number of lesions.

DWI, diffusion-weighted imaging; DWI_{800}, *b* -value of 800 s/mm^2 DWI; DWI_{1500}, *b* -value of 1500 s/mm^2 DWI.

DWI visibility was scored on a 5-point scale (0–4) by each reader independently (0 = poor to 4 = excellent).
